# Defensive Functioning of Individuals Diagnosed With Gender Dysphoria at the Beginning of Their Hormonal Treatment

**DOI:** 10.3389/fpsyg.2021.665547

**Published:** 2021-08-10

**Authors:** Guido Giovanardi, Marta Mirabella, Mariagrazia Di Giuseppe, Francesco Lombardo, Anna Maria Speranza, Vittorio Lingiardi

**Affiliations:** ^1^Department of Dynamic and Clinical Psychology, and Health Studies, “Sapienza” University of Rome, Rome, Italy; ^2^Department of Surgical, Medical and Molecular Pathology, Critical and Care Medicine, University of Pisa, Pisa, Italy; ^3^Laboratory of Seminology - Sperm Bank “Loredana Gandini”, Department of Experimental Medicine, “Sapienza” University of Rome, Rome, Italy

**Keywords:** defense mechanisms, gender dysphoria, transgender, personality, body satisfaction

## Abstract

Defense mechanisms are relevant indicators of psychological functioning and vulnerability to psychopathology. Their evaluation can unveil individuals' unconscious strategies for mediating reactions to emotional conflict and external stressors. At the beginning of their journey toward gender reassignment, individuals diagnosed with gender dysphoria (GD) may experience conflict and stressful experiences that trigger a wide range of defense mechanisms. Mature defenses may strengthen these individuals as they travel along this important path, while neurotic and immature defenses may exacerbate their body dissatisfaction (BD) and hinder their processing of change. Only a few studies have investigated self-reported defensive functioning in transgender people, finding a higher frequency of maladaptive defense mechanisms relative to controls. The present study was the first to apply an in-depth clinician-rated tool to assess the entire hierarchy of defense mechanisms within a sample of transgender people. Defensive functioning and personality organization were assessed in 36 individuals diagnosed with GD (14 trans women, 22 trans men, mean age 23.47 years), using the Defense Mechanisms Rating Scales (Perry, 1990) and the Shedler-Westen Assessment Procedure-200 (Shedler et al., 2014). Body uneasiness was assessed using the Body Uneasiness Test (BUT; Cuzzolaro et al., 2006). The findings showed that defensive functioning correlated positively with healthy personality functioning and negatively with BD. Compared to cisgender controls, participants with GD who presented greater defensive functioning were found to be more immature and to demonstrate significant differences in many levels of functioning. The clinical implications of the results suggest that psychological interventions aimed at improving defensive functioning in individuals with GD will be important in helping them manage the challenges posed by their gender transition.

## Introduction

Gender dysphoria GD; American Psychiatric Association. (2013) is a condition in which individuals experience distress due to an incongruence between their gender identity (or experienced/expressed gender) and the gender that was assigned to them at birth^1^. Over the past decade, GD has received increased research attention, and it is now considered a multi-factorial construct integrating biological, psychological, and social factors (De Vries and Cohen-Kettenis, 2012). Research has shown (for a review, see Dhejne et al., 2016) that the population of individuals with GD is heterogenous and vulnerable to several psychological challenges. In particular, recent studies have highlighted the risk for individuals with GD to suffer from several Axis I psychiatric disorders, such as depression, anxiety, and substance disorders (de Freitas et al., 2020). Mixed findings have been reported for Axis II personality disorders (PDs), with prevalence rates for this population ranging from 4.3% (Fisher et al., 2013) to 81.4% (Mazaheri Meybodi et al., 2014). Several studies involving transgender youth have highlighted the risk for this population of developing eating disorders or eating disorder symptoms (e.g., Feder et al., 2017), and for engaging in self-harming behavior and suicidal ideation and attempts (e.g., Aitken et al., 2016). Furthermore, recent studies have identified a potential link between GD and autism spectrum disorders (Hisle-Gorman et al., 2019; Warrier et al., 2020); however, this association is highly debated by experts in the field (e.g., Turban and van Schalkwyk, 2018).

As several studies have shown, body dissatisfaction (BD), which consists of negative feelings toward one's body and a negative evaluation of one's appearance, may be a key factor in the development of psychopathology (Bandini et al., 2013). Indeed, research (Vocks et al., 2009; Couturier et al., 2015; Witcomb et al., 2015; Becker et al., 2016; Jones et al., 2016; Mirabella et al., 2020) has reported that BD in individuals with GD extends beyond non-sexual body parts and, therefore, represents a significant source of suffering. Moreover, many studies have noted that the distress associated with BD may be increased in transgender people, due to discrimination and stigma in their life contexts (e.g., Clements-Nolle et al., 2006; Ristori and Steensma, 2016; Giovanardi et al., 2018, 2020a; Fortunato et al., 2020).

Gender-affirming treatments (e.g., social transition, whereby an individual adopts a name, pronoun, clothing, and hairstyle associated with their affirmed gender; Olson-Kennedy, 2016; Olson-Kennedy et al., 2016; Sherer, 2016) and hormonal and surgical interventions to modify the body according to one's affirmed gender have been found to significantly improve transgender people's mental health and well-being (Coleman et al., 2012). Recent reviews (Costa and Colizzi, 2016; Nguyen et al., 2018) have demonstrated that individuals with GD who have access to gender-affirming treatments present improvements in mental health outcomes and psychological well-being, including lower levels of anxiety and depression, perceived and social distress, personality-related psychopathology, suicidality, and higher quality of life, self-esteem, and body satisfaction. However, as several psychological guidelines and research studies underline (Bockting et al., 2006; Coleman et al., 2012; Giovanardi et al., 2019), gender transition and hormonal therapy can affect mood and PDs both positively and negatively (Matthys et al., 2021).

In this regard, the latest version of the Standards of Care for the Health of Transsexual, Transgender, and Gender Non-conforming People (SOC-7; Coleman et al., 2012), by the World Professional Association for Transgender Health (WPATH), and the Guidelines for Psychological Practice with Transgender and Gender Non-conforming People (American Psychiatric Association., 2015), published by the American Psychological Association (APA), underline the importance of adopting a multidisciplinary approach for the care of transsexual, transgender, and gender non-conforming people. The guidelines encourage the use of both physical (e.g., primary care, gynecologic and urologic care, reproductive options) and mental health support (e.g., assessment, counseling, psychotherapy) to maximize transgender people's overall health, psychological well-being, and self-fulfillment. Nonetheless, research on the protective and predictive factors of psychologically positive outcomes within gender transitions is scarce (Dhejne et al., 2016). In particular, the role of defense mechanisms, which are important mediators of psychological adjustment (Perry et al., 2019), is understudied in this population.

The DSM-5 conceptualizes defense mechanisms as “mechanisms that mediate the individual's reaction to internal or external stressors” (American Psychiatric Association., 2013). Such mechanisms are automatic processes that operate partially or wholly outside of an individual's awareness (Cramer, 1998). However, they may be identified in conversation by presenting an apparent incongruity with the outward meaning of the communication (Perry, 2014). Defense mechanisms can be both healthy and psychopathological, and they have been organized into a hierarchy based on their defensive function and level of adaptation (American Psychiatric Association., 1994; Vaillant, 1995). According to the gold-standard theoretical approach to the study of defense mechanisms (Vaillant, 1992, 2020; Perry, 2014), the Defense Mechanisms Rating Scales (DMRS; Perry, 1990) were developed to provide a valid and reliable observer-rated qualitative and quantitative assessment of 30 defense mechanisms, organized into 7 defense levels, 3 defensive categories, and an index of Overall Defensive Functioning (ODF). The ODF represents an overall summary measure, indicating the subject's level of defensive maturity (Perry and Bond, 2012).

Research has demonstrated the importance of the systematic assessment of defense mechanisms (Vaillant, 2020; Tanzilli et al., 2021). For instance, mature defenses (e.g., anticipation, humor, self-assertion) mitigate negative emotions and representations associated with conflict and distress (MacGregor and Olson, 2005; Martino et al., 2020), whereas immature defenses (e.g., splitting, denial, passive aggression) are linked to maladaptive personality traits at the base of several forms of psychopathology (Zimmerman et al., 2019; Boldrini et al., 2020; Perry et al., 2020). Several studies have shown that defense styles contribute significantly to individual differences in responses to stressful environments (Vaillant, 1992; Schulz et al., 2005; Cramer, 2006; Prout et al., 2020; Conversano, 2021; Di Giuseppe et al., 2021). Individuals with GD may be subject to numerous stressful events, due to social discrimination and stigma. The gender transition, itself, entailing massive changes to the body and many aspects of psychological functioning (e.g., emotion regulation), may produce a further risk factor for psychological adaptation.

As recommended by the international guidelines (Coleman et al., 2012), transgender people should have access to psychological resources both during and after their gender transition, to help them cope with any side effects of their treatment and to support them in adapting to their new reality. In this regard, we believe that defense mechanisms may be useful indexes of flexibility in individuals who are facing this journey, as well as useful prognostic variables to assess and promote in psychological counseling. Use of immature defenses (e.g., splitting, acting out) may hinder an individual's capacity to process changes, at both a physical and a psychological level. Moreover, use of particular defenses (e.g., projection, dissociation) might be associated with a significant level of BD. Lemma (2012, 2013) focused on the “embodied self” of transgender people and their “need to be seen” by caregivers and others not as “perverse,” but as “incongruent” —mirroring their felt “incongruence at the level of the body” (Lemma, 2013, p. 94). Moreover, as the psychoanalyst Saketopoulou (2014) noted, for transgender people, the ability to reflect, understand, and mentalize their body reality—in other words, the use of mature defenses (e.g., self-observation)—is key to achieving satisfying outcomes from a gender transition.

Despite research advances, literature on the defensive functioning of transgender people remains scarce. Lobato et al. (2009), using the self-report Defense Style Questionnaire (DSQ, Bond et al., 1983), investigated defenses in a sample of 32 trans women before and after gender reassignment, finding no significant differences 1 year post-surgery. However, the study lacked a control group, and thus the maturity of the defensive array could not be measured. Two studies (Sundbom et al., 1995; Sundbom and Bodlund, 1999) used a projective test, the Defense Mechanism Test, comparing patients with gender identity disorder (GID; American Psychiatric Association., 2000) to borderline patients and controls, finding higher frequencies of projection and introjection defenses in the GID sample. Finally, Prunas et al. (2014) used the self-report Response Evaluation Measure-71 (REM-71; Steiner et al., 2001) with a sample of 104 trans women and 36 trans men, compared to cisgender male and female controls, finding more maladaptive defensive functioning in trans women (but not trans men) compared to both control groups, including a higher use of immature defenses, such as projection, splitting, omnipotence, and fantasy.

Since defense mechanisms operate partially or wholly outside of awareness, self-report measures are limited to rating only their conscious correlates (Bond, 2004). Projective methods, on their part, have shown a lack of measurement validity on the entire hierarchy of defenses (Cramer, 1991). In light of the unconscious, dynamic, and functional nature of defense mechanisms, observer-rated instruments, applied in a clinical situation, are optimal for identifying when a defense is being used, and for what function (Perry and Ianni, 1998).

The present study aimed at analyzing the defensive functioning of individuals with GD and its association with personality adjustment and body satisfaction, in comparison to that of cisgender controls. Our first hypothesis was that higher ODF and greater use of mature defenses would be associated with higher personality functioning and lower body satisfaction. Conversely, lower ODF and greater use of immature defenses would be associated with lower personality functioning and BD. Our second hypothesis was that individuals with GD would show lower defensive functioning compared to cisgender controls. Finally, our third hypothesis was that certain defense mechanisms would differentiate individuals with GD from cisgender controls, with trans women presenting lower defensive adjustment.

## Methods

### Participants

The sample consisted of 36 adult participants, composed of 14 trans women and 22 trans men; mean age was 23.47 years (*SD* = 8.11). All participants had been diagnosed with GD in a specialized center in Rome, Italy, and were at stage T0 of hormonal therapy (waiting to start). They were recruited from the endocrinology unit of the Policlinico Umberto I Hospital of Rome. All participants declared an early onset of GD (during first or middle childhood, all before puberty). Two age-matched control groups (with the same mean age and standard deviation to the trans women and trans men, respectively), composed of 14 cisgender females and 22 cisgender males, were also extracted from a community sample analyzed in a previously published research project (Di Giuseppe et al., 2020). The experimental and control samples shared similar demographic characteristics, including a medium/high level of education, no marriage, and no children (Table 1). The study was approved by the Ethical Committee of the Department of Dynamic and Clinical Psychology, Sapienza University of Rome, Italy. All subjects provided written informed consent to participate.

**Table 1:** Sociodemographic characteristics of the samples (*N* = 72).

	**Transgender sample (** ***N*** **=** **36)**			**Cisgender controls (** ***N*** **=** **36)**		
	**Trans men (*N* = 22) (%)**	**Trans women (*N* = 14) (%)**	**Total (%)**	**Cis females (*N* = 22) (%)**	**Cis males (*N* = 14) (%)**	**Total (%)**
Age group						
<20	45	43	44	45	43	44
20–30	45	43	44	45	43	44
>30	9	14	11	9	14	11
Marital status						
Single	68	64	67	14	36	22
In a relationship	32	36	33	86	64	78
Level of education						
High School	15	29	31	9	–	5
College	64	57	61	64	71	67
Academic degree	5	14	8	27	29	28
Professional status						
Students	64	43	56	59	57	58
Employed	27	36	30	18	29	22
Unemployed	9	21	14	23	14	20

### Measures

#### Defense Mechanisms Rating Scale

The Defense Mechanisms Rating Scale (DMRS) (Perry, 1990) is an observer-based measure that assesses defense mechanisms from verbatim transcripts of clinical interviews or therapy sessions. The measure provides definitions, functions, and assessment procedures for 30 defense mechanisms, which are hierarchically organized into 7 defense levels and 3 defensive categories (see Table 2). The description for each defense includes examples of possible and certain uses, as well as a list of neighboring defenses to support differential analyses with respect to other defensive phenomena. The DMRS offers quantitative scores for: (1) Overall Defensive Functioning (ODF), representing a summary index of overall defensive adaptiveness, calculated by taking the average level of each defense score, weighted by its place in the hierarchy, yielding a score ranging from 1 (lowest) to 7 (highest); (2) 7 defense level scores, representing the proportional scores of each defense level, respectively; and (3) 30 individual defense scores, representing the proportional scores of each defense mechanism, respectively, calculated by dividing the occurrence of each defense in the transcript by the total instances of all defense mechanisms (Di Giuseppe et al., 2019). The convergent and discriminant validity of the DMRS is good for the overall hierarchy of defense mechanisms (Perry and Høglend, 1998), and inter-rater reliability between trained raters is high for the ODF and defense levels (intraclass *R* > 0.80) (Perry and Henry, 2004). In the present study, the interclass correlation (ICC) between two trained raters was calculated on six cases, resulting in a mean value of 0.76.

**Table 2:** Hierarchical organization of defense mechanisms in DMRS-based measures.

	**Defensive category**	**Defense level**	**Defense mechanism**
Overall Defensive Functioning (ODF)	Mature	High adaptive	Affiliation
			Altruism
			Anticipation
			Humor
			Self-assertion
			Self-observation
			Sublimation
			Suppression
	Neurotic	Obsessional	Intelletualization
			Isolation of affect
			Undoing
		Neurotic^b^	Displacement
			Dissociation
			Reaction formation
			Repression
	Immature^a^	Minor image-distorting	Devaluation
			Idealization
			Omnipotence
		Disavowal	Denial
			Projection
			Rationalization
			Autistic fantasy
		Major image-distorting	Projective identification
			Splitting of self-image
			Splitting of other's image
		Action	Acting out
			Help-rejecting complaining
			Passive aggression

*This table reports on previously published data (Di Giuseppe et al., 2020)*.

a *The immature category includes the categories of depressive and other immature (or non-depressive) defenses. The depressive category includes all action and major image-distorting defenses, plus projection, and devaluation. The other immature category includes autistic fantasy, rationalization, denial, omnipotence, and idealization*.

b *The neurotic defense level includes two sublevels of hysterical and other neurotic defenses. Hysterical defenses include repression and dissociation, while other neurotic defenses include displacement and reaction formation*.

#### Shedler Westen Assessment Procedure-200

The Shedler-Westen Assessment Procedure-200 (SWAP-200; Westen and Shedler, 1999a,b; Shedler et al., 2014) is a well-established and widely used psychometric procedure that was designed to provide a comprehensive assessment of personality and personality pathology. It consists of 200 personality-descriptive statements written in straightforward, experience-near language, to allow it to be used by clinicians with various theoretical orientations and levels of experience. The instrument utilizes a Q-sort method, which requires raters to sort items into eight categories, ranging from not descriptive to most descriptive of the individual, in order to comply with the fixed distribution. SWAP-200 PD scores, corresponding to 10 PD scales that are clinical prototypes of the DSM–IV–TR (American Psychiatric Association., 2000) and DSM−5 (American Psychiatric Association., 2013) PDs, produce a nomothetic diagnosis. Furthermore, the measure generates a healthy functioning score, reflecting clinicians' consensual understanding of the subject's adaptive personality functioning (Westen and Shedler, 1999a). The SWAP-200 has been shown to have very good validity and reliability, both with clinicians who have not been trained in using the instrument (Westen and Shedler, 1999a,b; Cogan and Porcerelli, 2004; Shedler and Westen, 2004; Blagov et al., 2012) and with clinicians who have received instrumental training (Bradley et al., 2007). The SWAP-200 has been used in previous studies involving transgender people (Lingiardi and Giovanardi, 2017; Lingiardi et al., 2017; Giovanardi et al., 2020b), and it has been shown to be clinically helpful in identifying personality subtypes within this population (Lingiardi et al., 2017) and in other clinical populations (e.g., Powers and Westen, 2009; Huprich et al., 2013; Muzi et al., 2020, 2021). In the present study, we used only the High-Functioning subscale, to correlate with the ODF score from the DMRS.

#### Body Uneasiness Test

The Body Uneasiness Test (BUT) (Cuzzolaro et al., 2000) is a self-report questionnaire that examines body shape and/or weight dissatisfaction, specific worries regarding particular body parts, avoidant and compulsive self-monitoring behaviors, feelings of detachment and estrangement toward one's own body, and body experiences and body image concerns. The measure comprises two parts. First, BUT-A consists of 34 items exploring body image concerns. Item scores are combined into a Global Severity Index (GSI), which is designed to assess a general level of body uneasiness, and five subscales, resulting from a factorial analysis (Cuzzolaro et al., 2000, 2006): Weight Phobia (fear of being or becoming fat), Body Image Concerns (worries related to physical appearance), Avoidance (avoidance behaviors related to body image), Compulsive Self-Monitoring (CSM; compulsive checking of physical appearance), and Depersonalization (feelings of detachment and estrangement toward the body). The second part of the measure, BUT-B, consists of 37 items exploring dissatisfaction with specific body parts (e.g., mouth, mustache, skin). The BUT-B produces two separate scores: a Positive Symptom Total (PST), which consists of the number of symptoms rated higher than 0, and a Positive Symptom Distress Index (PSDI), representing the average rating of those items constituting the PST. The present study utilized only the GSI. A GSI ≥ 1.2 is widely used as an index of clinically relevant discomfort with one's own body (Capoccia et al., 2015).

### Procedure

Participants were first asked to complete the BUT, then they were interviewed using the Clinical and Diagnostic Interview (CDI; Westen and Muderrisoglu, 2003)—a clinical interview that takes 2–3 h to administer, investigating personal history, affects, relationships, behaviors, affective states, emotion regulation processes, cognitive patterns, and history of symptoms and concerns, including severity, frequency, and duration. Each interview was recorded and transcribed. Clinical and Diagnostic Interview transcripts were rated by trained and reliable raters using the DMRS (Perry, 1990) and SWAP-200 (Shedler et al., 2014). Each evaluation was conducted blind and independent from the others, so no rater coded more than one measure for any single participant.

## Data Analysis

SPSS version 25 (IBM, Armonk, NY, United States) was used for the analyses. Bivariate correlations (Pearson's *r*, two-tailed) were calculated to study the relationship between defensive functioning (as assessed by the DMRS), personality functioning (as assessed by the SWAP-200), and body uneasiness in the experimental sample (*N* = 36). One-way analyses of variance (ANOVAs) were run to compare DMRS scores between the experimental and control groups (*N* = 72). A *post-hoc* Bonferroni test was applied to the ANOVAs to allow for multiple comparisons between groups.

## Results

### Relationship Between Defensive Functioning, Personality Functioning, and Body Uneasiness in Individuals With GD

The positive association between defensive functioning, personality functioning, and body satisfaction was tested using Pearson's correlations. The results showed that higher ODF scores and greater use of mature defenses correlated with higher personality functioning and greater body satisfaction. Conversely, lower ODF scores and greater use of immature defenses correlated with lower personality functioning and greater BD.

As Table 3 shows, overall defensive maturity (as indicated by the ODF score) and mature defenses were positively associated with a healthy personality (as indicated by the High Functioning Scale score) and negatively associated with BD (as indicated by the GSI). Moreover, use of immature defenses—particularly those in the depressive defensive category—was negatively correlated with a healthy personality, but unrelated to BD.

**Table 3:** Bivariate correlations between DMRS ODF, SWAP-200 high functioning scale, and BUT GSI scale (*N* = 36).

**DMRS scales and categories**	**SWAP-200**	**BUT**
	**High functioning scale**	**General index of severity**
ODF	0.681^**^	−0.357^*^
Mature defenses	0.624^**^	−0.414^*^
Neurotic defenses	n.s.	n.s.
Immature defenses	−0.583^**^	n.s.
Depressive defenses	−0.504^**^	n.s.
Non-depressive defenses	n.s.	n.s.

*DMRS, defense mechanisms rating scale; ODF, overall defensive functioning; SWAP-200, Shedler-Westen assessment procedure; BUT, body uneasiness test*.

* *p < 0.05*.

** *p < 0.01*.

### Comparisons of Defensive Functioning Between Transgender and Cisgender Groups

Differences in defensive functioning between the transgender and cisgender groups were tested using *T*-test analyses of the ODF, defensive categories, and defense levels. As presented in Table 4, individuals with GD obtained lower ODF scores (Δ = −0.49; *p* < 0.001) than cisgender controls. Both neurotic and immature defenses were used significantly more by transgender participants compared to controls, whereas mature defenses were used significantly less.

**Table 4:** *T*-tests comparing transgender sample and cisgender controls (*N* = 72).

	**Transgender sample (** ***N*** **=** **36)**		**Cisgender controls (** ***N*** **=** **36)**		**Δ Mean**	***t***	***p*-Value**
	**Mean**	***SD***	**Mean**	***SD***			
ODF	4.46	0.60	4.95	0.35	−0.49	−4.270	** <0.001**
Mature defenses	15.61	10.86	35.03	9.89	−19.42	−7.931	** <0.001**
Neurotic defenses	37.23	8.15	25.70	7.57	11.53	6.219	** <0.001**
Immature defenses	47.15	12.48	39.17	7.56	7.98	3.280	** <0.01**
Depressive defenses	24.00	11.12	19.16	6.49	4.84	2.257	** <0.05**
Non-depressive defenses	23.15	6.52	20.11	6.14	3.04	2.040	** <0.05**
High adaptive	15.61	10.86	35.03	9.89	−19.42	−7.931	** <0.001**
Obsessional	20.86	8.05	11.75	4.63	9.11	5.885	** <0.001**
Neurotic	16.37	5.59	13.95	4.75	2.42	1.978	0.052
Minor image-distorting	15.18	5.82	12.01	5.52	3.16	2.366	** <0.05**
Disavowal	16.72	6.63	15.21	4.53	1.51	1.132	0.261
Major image-distorting	3.11	2.63	5.74	3.53	−2.63	−3.583	** <0.001**
Action	12.15	7.43	6.22	3.21	5.93	4.398	** <0.001**

*Bold values indicate statistically significant*.

Deeper analyses of the differences between groups were conducted with respect to the seven defense levels, finding that transgender participants strongly differed from cisgender controls in their higher use of obsessional (Δ = 9.11; *p* < 0.001) and action (Δ = 5.93; *p* < 0.001) defenses and their lower use of high-adaptive (Δ = −19.42; *p* < 0.001) and major image-distorting (Δ = −2.63; *p* < 0.001) defenses. Transgender participants also demonstrated marginally higher use (*p* < 0.05) of neurotic and minor image-distorting defenses.

### Comparisons of Individual Defenses Between Subgroups (Trans Women vs. Trans Men vs. Cisgender Females vs. Cisgender Males)

Deeper analyses of the differences between transgender (trans women and trans men) and control (cisgender females and cisgender males) subgroups were achieved by investigating each subgroup's characteristic use of individual defense mechanisms.

#### Trans Women vs. Controls

Table 5 displays the results of the ANOVA and *post-hoc* Bonferroni tests comparing trans women with both control subgroups. Trans women showed strongly significantly lower scores on the ODF and several high-adaptive defenses, such as sublimation, humor, and anticipation, compared to both female and male cisgender participants. With respect to the mature defense of altruism, trans women produced a strongly significantly lower score (*p* < 0.001) than cisgender males, but not cisgender females. With regard to suppression and self-observation, trans women generated significantly lower scores than cisgender females (*p* < 0.01), but only marginally significantly lower scores than cisgender males (*p* < 0.05). Moreover, they scored significantly higher than both control subgroups on undoing and passive aggression, and significantly higher than only cisgender males on rationalization. Finally, trans women produced higher scores on projection than both control subgroups, and higher scores on repression than cisgender males.

**Table 5:** Analyses of variance (ANOVAs with Bonferrroni *post-hoc* tests) between trans women and controls (*N* = 50).

	**Trans women**	**Cisgender male**	**Cisgender female**	***F***	***p*-Value**
	**(*N* = 14)**	**controls (*N* = 22)**	**controls (*N* = 14)**		
ODF	4.27 (0.51)	5.04 (0.38)^†††^	4.89 (0.32)^†††^	15.358	** <0.001**
Suppression	0.77 (1.00)	3.36 (2.43)^†^	3.30 (2.58)^††^	6.694	** <0.001**
Sublimation	0.70 (1.05)	4.36 (2.91)^†††^	3.03 (2.18)^††^	10.050	** <0.001**
Self-observation	3.86 (3.26)	6.87 (2.93)^†^	7.76 (3.04)^††^	7.079	** <0.01**
Self-assertion	3.33 (2.99)	4.48 (2.19)	4.36 (2.76)	0.828	0.443
Humor	1.19 (1.76)	6.28 (3.92)^††^	5.89 (4.91)^††^	7.509	** <0.001**
Anticipation	0.20 (0.52)	2.77 (2.43)^††^	2.44 (2.03)^††^	8.067	** <0.001**
Altruism	0.38 (0.63)	5.03 (3.50)^†††^	2.64 (2.94)	10.284	** <0.001**
Affiliation	2.04 (2.03)	3.89 (3.64)	4.18 (3.08)	2.322	0.109
Isolation	2.27 (2.73)	2.92 (2.78)	2.38 (2.48)	0.250	0.780
Intellectualization	6.74 (3.97)	4.67 (3.76)	4.17 (2.49)	2.655	0.081
Undoing	11.05 (3.55)	4.60 (3.56)^***^	4.79 (3.42)^***^	16.560	** <0.001**
Repression	7.97 (3.54)	4.33 (3.17)^*^	5.91 (3.02)	4.514	** <0.05**
Dissociation	1.99 (2.63)	2.42 (3.51)	1.55 (1.50)	0.523	0.596
Reaction formation	1.49 (1.42)	3.09 (1.61)	2.88 (2.65)	2.523	0.091
Displacement	4.13 (2.47)	3.24 (2.14)	4.22 (2.82)	0.695	0.504
Deval. self	0.89 (1.29)	2.01 (2.04)	2.48 (2.34)	2.697	0.078
Deval. others	5.03 (2.90)	2.71 (2.88)	3.21 (2.87)	2.605	0.085
Ideal self	3.34 (2.52)	1.98 (2.00)	2.59 (3.11)	0.915	0.407
Ideal others	3.88 (2.73)	3.58 (1.82)	2.46 (2.34)	1.887	0.163
Omnipotence	2.09 (2.29)	1.99 (1.69)	1.18 (1.69)	1.285	0.308
Denial	2.65 (1.93)	2.83 (2.36)	2.78 (1.88)	0.032	0.969
Rationalization	11.31 (4.27)	6.25 (4.21)^**^	8.81 (4.09)	5.136	** <0.01**
Projection	3.87 (2.85)	1.75 (2.43)^*^	1.87 (1.67)^*^	4.121	** <0.05**
Autistic fantasy	1.86 (1.83)	3.49 (1.88)	2.27 (1.98)	2.827	0.069
Splitting self	0.91 (1.31)	1.72 (2.08)	1.73 (1.62)	1.178	0.317
Splitting others	1.63 (1.37)	2.00 (2.50)	2.42 (2.24)	0.606	0.550
Projective ident.	1.56 (1.22)	1.43 (1.44)	2.09 (2.16)	0.721	0.492
Passive aggression	8.12 (4.43)	1.74 (2.17)^***^	2.77 (2.37)^***^	18.580	** <0.001**
HRC	3.10 (3.63)	2.01 (2.15)	1.59 (1.55)	1.645	0.204
Acting out	1.65 (1.89)	2.30 (2.22)	1.94 (1.77)	0.394	0.676

*** *Indicates a strongly significantly higher score for trans women relative to male and female controls (p < 0.001)*.

** *Indicates a significantly higher score for trans women relative to male and female controls (p <0.01)*.

* *Indicates a marginally significantly higher score for trans women relative to male and female controls (p < 0.05).*

††† *Indicates a strongly significantly lower score for trans women relative to male and female controls (p < 0.001)*.

†† *Indicates a significantly lower score for trans women relative to male and female controls (p < 0.01).*

† *Indicates a marginally significantly lower score for trans women relative to male and female controls (p < 0.05). Bold values indicate statistically significant*.

#### Trans Men vs. Controls

Table 6 presents the results of the ANOVA and *post-hoc* Bonferroni tests comparing trans men and both control subgroups. Trans men generated only marginally lower ODF scores than cisgender males, whereas their scores were not significantly different from those of cisgender females. With respect to high-adaptive defenses, trans men produced significantly lower scores on suppression, sublimation, humor, and anticipation than both control subgroups (*p* < 0.001), and significantly lower scores on *altruism* (*p* < 0.001) and self-observation (*p* < 0.05) than cisgender males and females, respectively. Conversely, trans men scored significantly higher than both control subgroups on several defense mechanisms, including undoing, repression, and passive aggression. Moreover, *post-hoc* tests showed that trans men showed higher use of idealization of others-image (*p* < 0.05) and lower use of projective identification (*p* < 0.01) than cisgender females. Finally, they reported strongly significantly lower scores on autistic fantasy compared to cisgender males (*p* < 0.001), while only marginally significantly lower scores on this defense mechanism relative to cisgender females (*p* < 0.05).

**Table 6:** Analyses of variance (ANOVAs with Bonferroni *post-hoc* tests) between trans men and controls (*N* = 58).

	**Trans men**	**Cisgender male controls**	**Cisgender female controls**	***F***	***p*-Value**
	**(*N* = 22)**	**(*N* = 22)**	**(*N* = 14)**		
ODF	4.58 (0.63)	5.04 (0.38)^†^	4.89 (0.32)	4.606	** <0.05**
Suppression	0.98 (1.52)	3.36 (2.43)^††^	3.30 (2.58)^††^	7.754	** <0.001**
Sublimation	0.76 (1.29)	4.35 (2.91)^†††^	3.03 (2.18)^††^	13.527	** <0.001**
Self-observation	4.87 (4.30)	6.87 (2.93)	7.76 (3.04)^†^	3.770	** <0.05**
Self-assertion	3.99 (4.31)	4.48 (2.19)	4.36 (2.76)	0.111	0.895
Humor	1.49 (2.36)	6.28 (3.92)^†††^	5.89 (4.91)^†††^	9.515	** <0.001**
Anticipation	0.37 (0.90)	2.77 (2.43)^†††^	2.44 (2.03)^†††^	10.164	** <0.001**
Altruism	0.88 (1.48)	5.02 (3.50)^†††^	2.64 (2.94)	10.447	** <0.001**
Affiliation	4.26 (2.82)	3.89 (3.64)	4.18 (3.08)	0.064	0.938
Isolation	4.51 (5.66)	2.92 (2.78)	2.38 (2.48)	1.609	0.209
Intellectualization	6.16 (5.15)	4.67 (3.76)	4.17 (2.49)	1.449	0.244
Undoing	10.70 (4.02)	4.60 (3.56)^***^	4.79 (3.42)^***^	17.959	** <0.001**
Repression	10.23 (4.76)	4.33 (3.17)^***^	5.91 (3.02)^***^	12.177	** <0.001**
Dissociation	2.26 (2.44)	2.42 (3.51)	1.55 (1.50)	0.696	0.503
Reaction formation	1.64 (2.16)	3.09 (1.61)	2.88 (2.65)	2.400	0.100
Displacement	2.75 (2.38)	3.24 (2.14)	4.22 (2.82)	1.935	0.154
Deval. self	2.23 (2.17)	2.01 (2.04)	2.48 (2.34)	0.202	0.818
Deval. others	4.31 (2.61)	2.71 (2.88)	3.21 (2.87)	1.617	0.208
Ideal self	2.73 (2.51)	1.98 (2.00)	2.59 (3.11)	0.365	0.696
Ideal others	4.75 (4.03)	3.58 (1.82)	2.46 (2.34)^*^	3.170	** <0.05**
Omnipotence	1.13 (1.96)	1.99 (1.69)	1.18 (1.69)	1.129	0.331
Denial	3.18 (3.46)	2.83 (2.36)	2.78 (1.88)	0.136	0.873
Rationalization	9.18 (3.59)	6.25 (4.21)	8.81 (4.09)	2.628	0.081
Projection	1.55 (2.0)	1.75 (2.43)	1.87 (1.67)	0.149	0.862
Autistic fantasy	0.93 (1.52)	3.49 (1.88)^†††^	2.27 (1.98)^†^	9.022	** <0.001**
Splitting self	0.88 (1.83)	1.72 (2.08)	1.73 (1.62)	1.463	0.240
Splitting others	1.07 (1.10)	2.00 (2.50)	2.42 (2.24)	2.696	0.076
Projective ident.	0.53 (1.07)	1.43 (1.44)	2.09 (2.16)^††^	4.974	** <0.01**
Passive aggression	7.86 (6.66)	1.74 (2.17)^***^	2.77 (2.37)^***^	10.430	** <0.001**
HRC	1.54 (1.96)	2.01 (2.15)	1.59 (1.55)	0.304	0.739
Acting out	2.27 (2.81)	2.30 (2.22)	1.94 (1.77)	0.150	0.861

*** *Indicates a strongly significantly higher score for trans men relative to male and female controls (p < 0.001)*.

* *Indicates a marginally significantly higher score for trans men relative to male and female controls (p < 0.05)*.

††† *Indicates a strongly significantly lower score for trans men relative to male and female controls (p < 0.001)*.

†† *Indicates a significantly lower score for trans men relative to male and female controls (p < 0.01)*.

† *Indicates a marginally significantly lower score for trans men relative to male and female controls (p < 0.05). Bold values indicate statistically significant*.

#### Trans Women vs. Trans Men

Table 7 presents the results of the comparisons between trans women and trans men. *T*-test analyses showed a certain degree of homogeneity in defensive functioning across these subgroups. The only differences detected were as follows: trans women produced significantly higher scores on projection (*p* < 0.01) and projective identification (*p* < 0.05), while trans men generated marginally significantly higher scores on affiliation and devaluation of self-image (both *p* < 0.05).

**Table 7:** *T*-tests comparing trans women and trans men (*N* = 36).

	**Transgender women (** ***N*** **=** **14)**		**Transgender men (** ***N*** **=** **22)**		**Δ Mean**	***F***	***p*-Value**
	**Mean**	***SD***	**Mean**	***SD***			
ODF	4.27	0.51	4.58	0.63	−0.31	−1.539	0.133
Suppression	0.77	1.00	0.98	1.52	−0.21	−0.448	0.657
Sublimation	0.70	1.05	0.76	1.29	−0.05	−0.129	0.898
Self–observation	3.86	3.26	4.87	4.30	−1.00	−0.745	0.461
Self–assertion	3.33	2.99	3.99	4.31	−0.67	−0.506	0.616
Humor	1.19	1.76	1.49	2.36	−0.29	−0.399	0.692
Anticipation	0.20	0.52	0.37	0.90	−0.17	−0.646	0.522
Altruism	0.38	0.63	0.88	1.48	−0.51	−1.415	0.167
Affiliation	2.04	2.03	4.26	2.82	−2.22	−2.552	** <0.05**
Isolation of affects	2.27	2.73	4.51	5.66	−2.24	−1.378	0.177
Intellectualization	6.74	3.97	6.16	5.15	0.58	0.357	0.723
Undoing	11.05	3.55	10.70	4.02	0.35	0.265	0.793
Repression	7.97	3.54	10.23	4.76	−2.26	−1.528	0.136
Dissociation	1.99	2.63	2.26	2.44	−0.27	−0.316	0.754
Reaction formation	1.49	1.42	1.64	2.16	−0.15	−0.228	0.821
Displacement	4.13	2.47	2.75	2.38	1.37	1.663	0.105
Devaluation of self-image	0.89	1.29	2.23	2.17	−1.34	−2.107	** <0.05**
Devaluation of others-image	5.03	2.90	4.31	2.61	0.73	0.780	0.441
Idealization of self-image	3.34	2.52	2.73	2.51	0.62	0.717	0.479
Idealization of others-image	3.88	2.73	4.75	4.03	−0.87	−0.709	0.483
Omnipotence	2.09	2.29	1.13	1.96	0.95	1.332	0.192
Denial	2.65	1.93	3.18	3.46	−0.53	−0.525	0.603
Rationalization	11.31	4.27	9.18	3.59	2.13	1.613	0.116
Projection	3.87	2.85	1.55	2.0	2.32	2.882	** <0.01**
Autistic fantasy	1.86	1.83	0.93	1.52	0.94	1.665	0.105
Splitting of self-image	0.91	1.31	0.88	1.83	0.03	0.045	0.965
Splitting of others-image	1.63	1.37	1.07	1.10	0.56	1.359	0.183
Projective identification	1.56	1.22	0.53	1.07	1.03	2.662	** <0.05**
Passive aggression	8.12	4.43	7.86	6.66	0.26	0.128	0.899
HRC	3.09	3.63	1.55	1.96	1.55	1.668	0.104
Acting out	1.66	1.89	2.27	2.81	−0.62	−0.722	0.475

*Bold values indicate statistically significant*.

## Discussion

The present study demonstrated the relationship between defensive functioning, personality adjustment, and body satisfaction in a sample of individuals with GD at the beginning of their hormonal therapy. Applying the gold-standard tool for assessing the entire hierarchy of defense mechanisms, the study described the characteristic defensive profiles of trans women and trans men, compared to their cisgender counterparts.

Our first hypothesis was fully confirmed, given that an association was found between defensive maturity, personality adjustment, and body satisfaction. According to the literature (Blagov and Westen, 2007; Russ et al., 2008; Di Lallo et al., 2009; Powers and Westen, 2009; Colli et al., 2014), use of mature defenses is associated with healthy personality functioning, which is a protective factor against the development of psychopathology (Bond and Perry, 2004). This finding might suggest that the use of mature defenses may be a protective factor against BD, which is a key factor in the distress suffered by transgender people and at the core of some associated conditions, including anxiety, depression, and eating disorders (e.g., Bandini et al., 2013). These findings suggest that defensive maturity should be considered as a particularly informative index for the psychological functioning of individuals with GD.

With regard to our second hypothesis, that individuals with GD would present lower defensive functioning relative to cisgender controls, the findings confirmed that transgender people were likely to use more neurotic and immature defenses as compared to their cisgender counterparts. Consistent with previous studies (Sundbom et al., 1995, Sundbom and Bodlund, 1999; Prunas et al., 2014), transgender participants presented lower ODF scores and less use of mature defenses than controls, suggesting that individuals with GD who have not yet begin gender-affirming hormonal treatment may be especially vulnerable to developing various forms of psychopathology (Dhejne et al., 2016). In particular, significant differences were found in trans men compared to cisgender females, with the former demonstrating greater use of obsessional and action defenses and less use of major image-distorting defenses. In terms of defensive functioning, these findings reflect transgender people's need to maintain distance from conflictual charged feelings (i.e., obsessional defenses) that they cannot fully elaborate, which may result in an aggressive attitude toward the self or other in an attempt to mitigate internal tension (i.e., action defenses). Despite transgender people's evident difficulty with body image, our participants seemed aware of their need to integrate their perceived gender with their assigned gender, leading to a reduction in their use of major image-distorting defenses.

The present findings appear somewhat controversial in light of previous studies (e.g., Prunas et al., 2014), which found several borderline or major image-distorting defenses in transgender samples. This difference might reflect methodological differences. The measure used in this study to assess defense mechanisms, the DMRS, is the most comprehensive available instrument (gold-standard), supporting the deep investigation of defense mechanisms and the interpretation of defensive functions related to the use of detected defenses. Different from other commonly used measures, the DMRS and related measures (Di Giuseppe et al., 2014, 2020) have the unique strength of mapping definitions and functions to the entire hierarchy of defense mechanisms (Vaillant, 1992; American Psychiatric Association., 1994), revealing the unconscious function behind certain defensive profiles.

Our third hypothesis, which anticipated a characteristic use of defense mechanisms in transgender people and greater use of immature defenses by trans women, was also confirmed. Several defense mechanisms contributed to a unique defensive profile in individuals with GD, including undoing and passive aggression. Moreover, trans women showed greater use of rationalization compared to cisgender males, whereas trans men showed less use of repression and projective identification compared to cisgender females and less use of autistic fantasy compared to cisgender males.

Passive aggression, which is an immature defense used to cover up feelings of resentment and hostility toward others with apparent over-compliance, appeared most when participants described a lack of perceived support from family, friends, and school and medical staff. This defense mechanism is typically used by individuals who have learned to expect punishment or dismissal from caregivers in response to their expressed needs. In the present study, passive aggressive narratives often entailed descriptions of the self as a martyr or someone who was not entitled to receive support and acceptance, leading to expressions of “turning against the self” (i.e., self-punishing or self-harming behavior).

Similarly, trans women's high use of rationalization—a disavowal defense activated to avoid feelings of guilt or shame by justifying actions or claiming that external factors impelled the subject's behavior—seemed to reflect the same underlying dynamics. Trans women usually demonstrated this defense mechanism when describing stressful experiences with their caregivers, which often contained naïve or bizarre explanations of their caregivers' behaviors. With respect to transgender participants' mature defenses, their relatively high use of undoing—an obsessional defense—suggests that they managed aggression toward others through the use of contradictory statements, in order to mitigate any expression of emotional needs.

Of note, this type of defensive functioning does not typically relate to identity problems, in the way that major and minor image distortion defenses do (Rosa et al., 2019). With respect to these latter defenses, our sample scored similar to controls—or even lower, as in the case of trans men's projective identification and autistic fantasy. It is possible to hypothesize that obsessive defenses may serve to isolate emotional contents and restrain cognitions to specific aspects of reality, such as dissatisfaction with one's body or the desire to undergo a gender transition. This profound uneasiness might be the bedrock for the use of action-type defenses, including passive aggression, which are typically practiced by individuals who were raised in a rejecting environment and never had the opportunity to express and regulate their anger and develop trust in significant others (Kramer et al., 2013; Perry et al., 2013).

Overall, our findings highlight that interpretations of GD as a severe “identity” disorder, as proposed by many psychoanalytic authors—often associating it with severe narcissistic disorders (Oppenheimer, 1991) or psychotic symptomatology (Chiland, 2000)—are shortsighted. Indeed, the defenses associated with these disorders do not seem to align with the general defensive functioning of our sample. Conversely, our findings support the idea of greater external sources of suffering linked to GD, connected to a lack of recognition and mirroring from one's environment, rather than self-image distortion. Transgender people's stressful lived experiences may polarize their defensive functioning toward a self-sacrificing dysregulation between their thoughts, feelings, and actions. In line with previous research on defense mechanisms in transgender people (Prunas et al., 2014), the present study found higher defensive functioning in trans men compared to trans women. In particular, trans men were more likely to devalue their qualities and turn to others for help or support, whereas trans women were more likely to project conflictual feelings, impulses, and thoughts and blame others for their emotional distress.

The present findings have several clinical implications. First, our results demonstrate that the assessment of defenses using the DMRS and related instruments may be helpful in predicting the maturity and flexibility of psychological organization in individuals at the beginning of their gender transition journey. Second, the findings highlight the need to support individuals with GD, who are more psychologically vulnerable than their cisgender counterparts, before, during, and after their gender transition. Several studies (Hoglend and Perry, 1998; Perry et al., 1998; Hersoug et al., 2002; Drapeau et al., 2003) have shown that effective psychological support may improve defensive functioning and thereby equip subjects with more protective factors to help them manage stressful conditions. In this regard, it is important to consider the role of psychological support for transgender people not as a mechanism for counteracting splitting or distorting self-image—as previously intended by many authors (e.g., Chiland, 2000)—but as one aimed at improving subjects' capacity to elaborate stressful life experiences and to mentalize the changes they experience in their body and mind during their gender transition (Saketopoulou, 2014). Finally, consistent with the recommendations of several international guidelines (e.g., Coleman et al., 2012), psychological support must be provided in the context of a multidisciplinary gender-affirming approach to care, whereby subjects are accompanied by a team of medical and psychological experts.

## Limitations and Future Directions

Despite its strengths, the present study also presented some limitations. First, the sample size was relatively small, and therefore generalization to the entire population of individuals with GD must be drawn with caution. Moreover, in our sample we found a medium-to-high level of education, and thus this study may have overlooked important aspects of transgender populations with lower level of education. Further research involving larger stratified samples should be pursued to confirm these findings. Second, due to the cross-sectional nature of the research, only exploratory analyses of associations between the studied variables were possible. Our transgender participants were all at the beginning of a process that would have had a deep impact on their life and were thus highly exposed to stress, which could have developed defense mechanisms. Longitudinal studies should be designed to gain insight on how defense mechanisms might impact the adjustment of transgender people to the entire gender transition process. Finally, the lack of information on psychiatric symptoms at the time of interview might have led us to overlook potentially significant factors in individual defensive functioning. Considering the predictive value of defensive functioning on mental health (Conversano and Di Giuseppe, 2021; Hersoug et al., 2021), future research should seek to produce a comprehensive assessment of psychological changes during gender transitions, both before and after hormone therapy and gender reassignment surgery, in order to better understand the impact of psychological and psychosocial factors on defensive functioning.

## Data Availability Statement

The raw data supporting the conclusions of this article will be made available by the authors, without undue reservation.

## Ethics Statement

The studies involving human participants were reviewed and approved by Department of Dynamic and Clinical Psychology, and Health Studies, Sapienza University of Rome, Italy. The patients/participants provided their written informed consent to participate in this study.

## Author Contributions

GG and VL conceived the research study. MM and FL contributed to data collection. MD performed DMRS ratings. GG performed data analyses and wrote the first draft of the manuscript. AS and VL contributed to the interpretation of the results. All authors critically reviewed the final draft of the manuscript.

## Conflict of Interest

The authors declare that the research was conducted in the absence of any commercial or financial relationships that could be construed as a potential conflict of interest.

## Publisher's Note

All claims expressed in this article are solely those of the authors and do not necessarily represent those of their affiliated organizations, or those of the publisher, the editors and the reviewers. Any product that may be evaluated in this article, or claim that may be made by its manufacturer, is not guaranteed or endorsed by the publisher.

## Footnotes

^1^In the present research, the sample was comprised of individuals who had received a diagnosis of GD; thus, the terminology used to describe our sample will be “individuals with GD” or, more generally, “transgender people.” The sample subgroups will be identified as “trans women” (assigned male at birth) and “trans men” (assigned female at birth).

## References

[B1] Aitken M. VanderLaan D. P. Wasserman L. Stojanovski S. Zucker K. J. (2016). Self-harm and suicidality in children referred for gender dysphoria. J. Am. Acad. Child Adolesc. Psychiatry 55, 513–520. 10.1016/j.jaac.2016.04.001 27238070

[B2] American Psychiatric Association. (1994). Diagnostic and Statistical Manual of Mental Disorders, 4th Edn. Arlington, VA: American Psychiatric Association.

[B3] American Psychiatric Association. (2000). Diagnostic and Statistical Manual of Mental Disorders, 4th Edn. Text Rev. Arlington, VA: American Psychiatric Association.

[B4] American Psychiatric Association. (2013). Diagnostic and Statistical Manual of Mental Disorders, 5th Edn. Arlington, VA: American Psychiatric Association.

[B5] American Psychiatric Association. (2015). Guidelines for psychological practice with transgender and gender nonconforming people. Am. Psychol. 70, 832–864. 10.1037/a0039906 26653312

[B6] Bandini E. Fisher A. D. Castellini G. Sauro C. L. Lelli L. Meriggiola M. C.. (2013). Gender identity disorder and eating disorders: similarities and differences in terms of body uneasiness. J. Sex. Med. 10, 1012–1023. 10.1111/jsm.12062 23347389

[B7] Becker I. Nieder T. O. Cerwenka S. Briken P. Kreukels B. P. Cohen-Kettenis P. T.. (2016). Body image in young gender dysphoric adults: a European multi-center study. Arch. Sex. Behav. 45, 559–574. 10.1007/s10508-015-0527-z 25836027

[B8] Blagov P. Westen D. (2007). Under the Axis II radar: clinically relevant personality constellations that escape DSM-IV diagnosis. J. Nerv. Ment. Dis. 195, 477–483. 10.1097/NMD.0b013e318064e824 17568295

[B9] Blagov P. S. Bi W. Shedler J. Westen D. (2012). The Shedler-Westen Assessment Procedure (SWAP): evaluating psychometric questions about its reliability, validity, and impact of its fixed score distribution. Assessment 19, 370–382. 10.1177/1073191112436667 22327208

[B10] Bockting W. O. Knudson G. Goldberg J. M. (2006). Counseling and mental health care for transgender adults and loved ones. Int. J. Transgend. 9, 35–82. 10.1300/J485v09n03_03

[B11] Boldrini T. Lo Buglio G. Giovanardi G. Lingiardi V. Salcuni S. (2020). Defense mechanisms in adolescents at high risk of developing psychosis: an empirical investigation. Res. Psychother. 23:456. 10.4081/ripppo.2020.456 32913831 PMC7451313

[B12] Bond M. (2004). Empirical studies of defense style: relationships with psychopathology and change. Harv. Rev. Psychiatry 12, 263–278. 10.1080/10673220490886167 15590575

[B13] Bond M. Gardner S. T. Christian J. Sigal J. J. (1983). Empirical study of self-rated defense styles. Arch. Gen. Psychiatry 40, 333–338. 10.1001/archpsyc.1983.01790030103013 6830412

[B14] Bond M. Perry J.–C. (2004). Long-term changes in defense styles with psychodynamic psychotherapy for depressive, anxiety and personality disorders. Am. J. Psychiatry 161, 1665–1671. 10.1176/appi.ajp.161.9.1665 15337658

[B15] Bradley R. Hilsenroth M. Guarnaccia C. Westen D. (2007). Relationship between clinician assessment and self-assessment of personality disorders using the SWAP-200 and PAI. Psychol. Assess. 19, 225–229. 10.1037/1040-3590.19.2.225 17563203

[B16] Capoccia D. Monaco V. Coccia F. Leonetti F. Cavaggioni G. (2015). Axis II disorders, body image and childhood abuse in bariatric surgery candidates. Clin. Ter. 166, e248–e253. 10.7417/T.2015.1868 26378757

[B17] Chiland C. (2000). The psychoanalyst and the transsexual patient. Int. J. Psychoanal. 81, 21–35. 10.1516/0020757001599483 10816842

[B18] Clements-Nolle K. Marx R. Katz M. (2006). Attempted suicide among transgender persons: the influence of gender-based discrimination and victimization. J. Homosex. 51, 53–69. 10.1300/J082v51n03_04 17135115

[B19] Cogan R. Porcerelli J. H. (2004). Personality pathology, adaptive functioning, and strengths at the beginning and end of psychoanalysis. J. Am. Psychoanal. Assoc. 52, 1230–1231.

[B20] Coleman E. Bockting W. Botzer M. Cohen-Kettenis P. DeCuypere G. Feldman J.. (2012). Standards of care for the health of transsexual, transgender, and gender-nonconforming people, version 7. Int. J. Transgend. 13, 165–232. 10.1080/15532739.2011.700873

[B21] Colli A. Tanzilli A. Dimaggio G. Lingiardi V. (2014). Patient personality and therapist response: an empirical investigation. Am. J. Psychiatry 171, 102–108. 10.1176/appi.ajp.2013.13020224 24077643

[B22] Conversano C. (2021). The psychodynamic approach during COVID-19 emotional crisis. Front. Psychol. 12:670196. 10.3389/fpsyg.2021.670196 33897574 PMC8062854

[B23] Conversano C. Di Giuseppe M. (2021). Psychological factors as determinants of chronic conditions: clinical and psychodynamic advances. Front. Psychol. 12:635708. 10.3389/fpsyg.2021.635708 33584488 PMC7876054

[B24] Costa R. Colizzi M. (2016). The effect of cross-sex hormonal treatment on gender dysphoria individuals' mental health: a systematic review. Neuropsychiatr. Dis. Treat. 12, 1953–1966. 10.2147/NDT.S95310 27536118 PMC4977075

[B25] Couturier J. Pindiprolu B. Findlay S. Johnson N. (2015). Anorexia nervosa and gender dysphoria in two adolescents. Int. J. Eat. Disord. 48, 151–155. 10.1002/eat.22368 25421316

[B26] Cramer P. (1991). The defense mechanism manual, in The Development of Defense Mechanisms, ed Cramer P. (New York, NY: Springer), 215–234. 10.1007/978-1-4613-9025-1

[B27] Cramer P. (1998). Coping and defense mechanisms: what's the difference? J. Pers. 66, 919–946. 10.1111/1467-6494.00037

[B28] Cramer P. (2006). Protecting the Self: Defense Mechanisms in Action. New York, NY: Guilford Press.

[B29] Cuzzolaro M. Vetrone G. Marano G. Battacchi M. (2000). Body uneasiness test, in Repertorio delle Scale di Valutazione in Psichiatria, Vol. 3, ed Conti L. (Firenze: Societa' Editrice Universo), 1759–1761.

[B30] Cuzzolaro M. Vetrone G. Marano G. Garfinkel P. E. (2006). The Body Uneasiness Test (BUT): development and validation of a new body image assessment scale. Eat. Weight Disord. 11, 1–13. 10.1007/BF03327738 16801740

[B31] de Freitas L. D. Léda-Rêgo G. Bezerra-Filho S. Miranda-Scippa Â. (2020). Psychiatric disorders in individuals diagnosed with gender dysphoria: a systematic review. Psychiatry Clin. Neurosci. 74, 99–104. 10.1111/pcn.12947 31642568

[B32] De Vries A. L. Cohen-Kettenis P. T. (2012). Clinical management of gender dysphoria in children and adolescents: the Dutch approach. J. Homosex. 59, 301–320. 10.1080/00918369.2012.653300 22455322

[B33] Dhejne C. van Vlerken R. Heylens G. Arcelus J. (2016). Mental health and gender dysphoria: a review of the literature. Int. Rev. Psychiatry 28, 44–57. 10.3109/09540261.2015.1115753 26835611

[B34] Di Giuseppe M. Gennaro A. Lingiardi V. Perry J. C. (2019). The role of defense mechanisms in emerging personality disorders in clinical adolescents. Psychiatry 82 128–142. 10.1080/00332747.2019.1579595 30925112

[B35] Di Giuseppe M. Nepa G. Prout T. A. Albertini F. Marcelli S. Orrù G.. (2021). Stress, burnout, and resilience among healthcare workers during the COVID-19 emergency: the role of defense mechanisms. Int. J. Environ. Res. Public Health. 18:5258. 10.3390/ijerph18105258 34069270 PMC8156145

[B36] Di Giuseppe M. Perry J. C. Lucchesi M. Michelini M. Vitiello S. Piantanida A.. (2020). Preliminary reliability and validity of the DMRS-SR-30, a novel self-report based on the Defense Mechanisms Rating Scales. Front. Psychiatry 11:870. 10.3389/fpsyt.2020.00870 33005160 PMC7479239

[B37] Di Giuseppe M. Perry J. C. Petraglia J. Janzen J. Lingiardi V. (2014). Development of a Q-sort version of the Defense Mechanism Rating Scales (DMRS-Q) for clinical use. J. Clin. Psychol. 70, 452–465. 10.1002/jclp.22089 24706519

[B38] Di Lallo J. J. Jones M. Westen D. (2009). Personality subtypes in disruptive adolescent males. J. Nerv. Ment. Dis. 197, 15–23. 10.1097/NMD.0b013e318192770c 19155805

[B39] Drapeau M. de Roten Y. Perry J. C. Despland J. N. (2003). A study of stability and change in defense mechanisms during a brief psychodynamic investigation. J. Nerv. Ment. Dis. 191, 496–502. 10.1097/01.nmd.0000082210.76762.ec 12972851

[B40] Feder S. Isserlin L. Seale E. Hammond N. Norris M. L. (2017). Exploring the association between eating disorders and gender dysphoria in youth. Eat. Disord. 25, 310–317. 10.1080/10640266.2017.1297112 28281883

[B41] Fisher A. D. Bandini E. Casale H. Ferrucio N. Meriggiola M. C. Gualerzi A.. (2013). Sociodemographic and clinical features of gender identity disorder: an Italian multicentric evaluation. J. Sex. Med. 10, 408–419. 10.1111/j.1743-6109.2012.03006.x 23171237

[B42] Fortunato A. Giovanardi G. Mirabella M. Di Ceglie D. Speranza A. M. Caviglia G.. (2020). Caring for gender diverse children and adolescents in Italy: a mixed-method investigation of clinicians' knowledge and approach to clinical practice. Clin. Child Psychol. Psychiatry 25, 1049–1067. 10.1177/1359104520925526 32498554

[B43] Giovanardi G. Fortunato A. Mirabella M. Speranza A. M. Lingiardi V. (2020a). Gender diverse children and adolescents in Italy: a qualitative study on specialized centers' model of care and network. Int. J. Environ. Res. Public Health 17:9536. 10.3390/ijerph17249536 33352745 PMC7766564

[B44] Giovanardi G. Morales P. Mirabella M. Fortunato A. Chianura L. Speranza A. M.. (2019). Transition memories: experiences of trans adult women with hormone therapy and their beliefs on the usage of hormone blockers to suppress puberty. J. Enodcrinol. Investig. 42, 1231–1240. 10.1007/s40618-019-01045-2 30953318

[B45] Giovanardi G. Mundo E. Lingiardi V. (2020b). Paola on the couch: the quest for feminine identity in an empirically supported psychoanalytic psychotherapy of a trans woman. Psychoanal. Psychol. 10.1037/pap0000330. [Epub ahead of print].

[B46] Giovanardi G. Vitelli R. Maggiora Vergano C. Fortunato A. Chianura L. Lingiardi V. (2018). Attachment patterns and complex trauma in a sample of adults diagnosed with gender dysphoria. Front. Psychol. 9:60. 10.3389/fpsyg.2018.00060 29449822 PMC5799708

[B47] Hersoug A.G. Wærsted M. Lau B. (2021) Defensive functioning moderates the effects of nondirective meditation. Front. Psychol. 12:629784. 10.3389/fpsyg.2021.629784 33584485 PMC7876444

[B48] Hersoug A. G. Monsen J. Havik O. D. Hoglend P. (2002). Quality of early working alliance in psychotherapy: diagnoses, relationship and intrapsychic variables as predictors. Psychother. Psychosom. 71, 18–27. 10.1159/000049340 11740165

[B49] Hisle-Gorman E. Landis C. A. Susi A. Schvey N. A. Gorman G. H. Nylund C. M.. (2019). Gender dysphoria in children with autism spectrum disorder. LGBT Health 6, 95–100. 10.1089/lgbt.2018.0252 30920347

[B50] Hoglend P. Perry J. C. (1998). Defensive functioning predicts improvement in treated major depressive episodes. J. Nerv. Ment. Dis. 186, 238–243. 10.1097/00005053-199804000-00006 9569892

[B51] Huprich S. K. DeFife J. Westen D. (2013). Refining a complex diagnostic construct: subtyping dysthymia with the Shedler-Westen Assessment Procedure-II. J. Affect. Disord. 186–192. 10.1016/j.jad.2013.09.008 24120405

[B52] Jones B. A. Haycraft E. Murjan S. Arcelus J. (2016). Body dissatisfaction, and disordered eating in trans people: a systematic review of the literature. Int. Rev. Psychiatry 28, 81–94. 10.3109/09540261.2015.1089217 26618239

[B53] Kramer U. de Roten Y. Perry J. C. Despland J.-N. (2013). Beyond splitting: observer-rated defense mechanisms in borderline personality disorder. Psychoanal. Psychol. 30, 3–15. 10.1037/a0029463

[B54] Lemma A. (2012). Research off the couch: re-visiting the transsexual conundrum. Psychoanal. Psychother. 26, 263–281. 10.1080/02668734.2012.732104

[B55] Lemma A. (2013). The body one has and the body one is: understanding the transsexual's need to be seen. Int. J. Psychoanal. 94, 277–292. 10.1111/j.1745-8315.2012.00663.x 23560903

[B56] Lingiardi V. Giovanardi G. (2017). Challenges in assessing personality of individuals with gender dysphoria with the SWAP-200. J. Endocrinol. Invest. 40, 693–703. 10.1007/s40618-017-0629-7 28238165

[B57] Lingiardi V. Giovanardi G. Fortunato A. Nassisi V. Speranza A. M. (2017). Personality and attachment in transsexual adults. Arch. Sex. Behav. 46, 1313–1323. 10.1007/s10508-017-0946-0 28210932

[B58] Lobato M. I. Koff W. J. Crestana T. Chaves C. Salvador J. Rodolpho Petry A.. (2009). Using the Defensive Style Questionnaire to evaluate the impact of sex reassignment surgery on defensive mechanisms in transsexual patients. Braz. J. Psychiatry 31, 303–306. 10.1590/S1516-44462009005000007 19838593

[B59] MacGregor M. W. Olson T. R. (2005). Defense mechanisms: their relation to personality and health. an exploration of defense mechanisms assessed by the defense-Q, in Advances in Psychology Research, Vol. 36, ed Columbus A. (Hauppauge, NY: Nova Science Publishers), 95–141.

[B60] Martino G. Caputo A. Bellone F. Quattropani M. C. Vicario C. M. (2020). Going beyond the visible in type 2 diabetes mellitus: defense mechanisms and their associations with depression and health-related quality of life. Front. Psychol. 11:267. 10.3389/fpsyg.2020.00267 32174865 PMC7054284

[B61] Matthys I. Defreyne J. Elaut E. Fisher A. D. Kreukels B. Staphorsius A.. (2021). Positive and negative affect changes during gender-affirming hormonal treatment: results from the European Network for the Investigation of Gender Incongruence (ENIGI). J. Clin. Med. 10:296. 10.3390/jcm10020296 33466910 PMC7829763

[B62] Mazaheri Meybodi A. Hajebi A. Ghanbari Jolfaie A. (2014). Psychiatric axis I comorbidities among patients with gender dysphoria. Psychiatry J. 2014:971814. 10.1155/2014/971814 25180172 PMC4142737

[B63] Mirabella M. Giovanardi G. Fortunato A. Senofonte G. Lombardo F. Lingiardi V.. (2020). The body I live in. Perceptions and meanings of body dissatisfaction in young transgender adults: a qualitative study. J. Clin. Med. 9:3733. 10.3390/jcm9113733 33233761 PMC7699932

[B64] Muzi L. Tieghi L. Franco A. Rugo M. Lingiardi V. (2021). The mediator effect of personality on the relationship between symptomatic impairment and treatment outcome in eating disorders. Front. Psychol. 12:688924. 10.3389/fpsyg.2021.688924 34276515 PMC8282821

[B65] Muzi L. Tieghi L. Rugo M. A. Lingiardi V. (2020). Personality as a predictor of symptomatic change in a residential treatment setting for anorexia nervosa and bulimia nervosa. Eat. Weight Disord. 26, 1195–1209. 10.1007/s40519-020-01023-1 33048329 PMC8062347

[B66] Nguyen H. B. Chavez A. M. Lipner E. Hantsoo L. Kornfield S. L. Davies R. D.. (2018). Gender-affirming hormone use in transgender individuals: impact on behavioral health and cognition. Curr. Psychiatry Rep. 20:110. 10.1007/s11920-018-0973-0 30306351 PMC6354936

[B67] Olson-Kennedy J. (2016). Mental health disparities among transgender youth: rethinking the role of professionals. JAMA Pediatr. 170, 423–424. 10.1001/jamapediatrics.2016.0155 26998945

[B68] Olson-Kennedy J. Cohen-Kettenis P. T. Kreukels B. P. Meyer-Bahlburg H. F. Garofalo R. Meyer W.. (2016). Research priorities for gender nonconforming/transgender youth: gender identity development and biopsychosocial outcomes. Curr. Opin. Endocrinol. Diabetes Obes. 23, 172–179. 10.1097/MED.0000000000000236 26825472 PMC4807860

[B69] Oppenheimer A. (1991). The wish for a sex change: a challenge to psychoanalysis? Int. J. Psychoanal. 72, 221–231. 1874585

[B70] Perry J. C. (1990). Defense Mechanism Rating Scales (DMRS), 5th Edn. Cambridge: Harvard School of Medicine.

[B71] Perry J. C. (2014). Anomalies and specific functions in the clinical identification of defense mechansims. J. Clin. Psychol. 70, 406–418. 10.1002/jclp.22085 24677150

[B72] Perry J. C. Banon E. Bond M. (2020). Change in defense mechanisms and depression in a pilot study of antidepressive medications plus 20 sessions of psychotherapy for recurrent major depression. J. Nerv. Mental Dis. 208, 261–268. 10.1097/NMD.0000000000001112 32221178

[B73] Perry J. C. Bond M. (2012). Change in defense mechanisms during long-term dynamic psychotherapy and five-years outcome. Am. J. Psychiatry 69, 916–925. 10.1176/appi.ajp.2012.11091403 22885667

[B74] Perry J. C. Henry M. (2004). Studying defense mechanisms in psychotherapy using the Defense Mechanism Rating Scales, in Defense Mechanisms: Theoretical, Research and Clinical Perspectives, eds Hentschel U. Smith G. Draguns J. G. Ehlers W. (Oxford: Elsevier Science Ltd), 165–192.

[B75] Perry J. C. Høglend P. (1998). Convergent and discriminant validity of overall defensive functioning. J. Nerv. Ment. Dis. 186, 529–535. 10.1097/00005053-199809000-00003 9741558

[B76] Perry J. C. Hoglend P. Shear K. Vaillant G. E. Horowitz M. J. Kardos M. E.. (1998). Field trial of a diagnostic axis for defense mechanisms for DSM–IV. J. Pers. Disord. 12, 56–68. 10.1521/pedi.1998.12.1.56 9573520

[B77] Perry J. C. Ianni F. (1998). Observer-rated measures of defense mechanisms. J. Pers. 66, 993–1024. 10.1111/1467-6494.00040

[B78] Perry J. C. Knoll M. Tran V. (2019). Motives, defenses, and conflicts in the dynamic formulation for psychodynamic psychotherapy using the idiographic conflict formulation method, in Case Formulation for Personality Disorders, ed Kramer U. (New York, NY: Elsevier), 203–224. 10.1016/B978-0-12-813521-1.00011-4

[B79] Perry J. C. Presniak M. D. Olson T. R. (2013). Defense mechanisms in schizotypal, borderline, antisocial, and narcissistic personality disorders. Psychiatry 76, 32–52. 10.1521/psyc.2013.76.1.32 23458114

[B80] Powers A. Westen D. (2009). Personality subtypes in patients with panic disorder. Compr. Psychiatry 50, 164–172. 10.1016/j.comppsych.2008.07.006 19216894

[B81] Prout T. A. Zilcha-Mano S. Aafjes-van Doorn K. Békés V. Christman-Cohen I. Whistler K.. (2020). Identifying predictors of psychological distress during COVID-19: a machine learning approach. Front. Psychol. 11:586202. 10.3389/fpsyg.2020.586202 33240178 PMC7682196

[B82] Prunas A. Vitelli R. Agnello F. Curti E. Fazzari P. Giannini F.. (2014). Defensive functioning in MtF and FtM transsexuals. Compr. Psychiatry 55, 966–971. 10.1016/j.comppsych.2013.12.009 24439558

[B83] Ristori J. Steensma T. D. (2016). Gender dysphoria in childhood. Int. Rev. Psychiatry 28, 13–20. 10.3109/09540261.2015.1115754 26754056

[B84] Rosa V. Tomai M. Lauriola M. Martino G. Di Trani M. (2019). Body mass index, personality traits, and body image in Italian pre-adolescents: an opportunity for overweight prevention. Psihologija 52–9-9. 10.2298/PSI181121009R

[B85] Russ E. Shedler J. Bradley R. Westen D. (2008). Refining the construct of narcissistic personality disorder: diagnostic criteria and subtypes. Am. J. Psychiatry 165, 1473–1481. 10.1176/appi.ajp.2008.07030376 18708489

[B86] Saketopoulou A. (2014). Mourning the body as bedrock. J. Am. Psychoanal. Assoc. 62, 773–806. 10.1177/0003065114553102 25277869

[B87] Schulz M. S. Waldinger R. J. Hauser S. T. Allen J. P. (2005). Adolescents' behavior in the presence of interparental hostility. Dev. Psychopathol. 17, 489–507. 10.1017/S0954579405050236 16761555 PMC1557645

[B88] Shedler J. Westen D. (2004). Refining personality disorder diagnosis: integrating science and practice. Am. J. Psychiatry 161, 1350–1365. 10.1176/appi.ajp.161.8.1350 15285958

[B89] Shedler J. Westen D. Lingiardi V. (2014). La Valutazione della Personalita' con la SWAP-200 [Personality Assessment with the SWAP-200]. Milano: Raffaello Cortina.

[B90] Sherer I. (2016). Social transition: supporting our youngest transgender children. Pediatrics 137:e20154358. 10.1542/peds.2015-4358 26921284

[B91] Steiner H. Araujo K. B. Koopman C. (2001). The Response Evaluation Measure (REM-71): a new instrument for the measurement of defenses in adults and adolescents. Am. J. Psychiatry 158, 467–473. 10.1176/appi.ajp.158.3.467 11229990

[B92] Sundbom E. Bodlund O. (1999). Prediction of outcome in transsexualism by means of the defense mechanism test and multivariate modelling: a pilot study. Percept. Mot. Skills 88, 3–20. 10.2466/pms.1999.88.1.3 10214627

[B93] Sundbom E. Bodlund O. Hojerback K. (1995). Object relations and defensive operations in transsexuals and borderline patients as measured by the Defense Mechanism Test. Nord. J. Psychiatry 49, 379–388. 10.3109/08039489509011931

[B94] Tanzilli A. Di Giuseppe M. Giovanardi G. Boldrini T. Caviglia G. Conversano C.. (2021). Mentalization, attachment, and defense mechanisms: a Psychodynamic Diagnostic Manual-2-oriented empirical investigation. Res. Psychother. 31–41. 10.4081/ripppo.2021.531 33937117 PMC8082535

[B95] Turban J. L. van Schalkwyk G. I. (2018). “Gender dysphoria” and autism spectrum disorder: is the link real? J. Am. Acad. Child Adolesc. Psychiatry 57, 8–9. 10.1016/j.jaac.2017.08.017 29301673

[B96] Vaillant G. E. (2020). Defense mechanisms, in Encyclopedia of Personality and Individual Differences, eds Zeigler-Hill V. Shackelford T. K. (Springer, Cham). 10.1007/978-3-319-24612-3_1372

[B97] Vaillant G. E. (1992). Ego Mechanisms of Defense: A Guide for Clinicians and Researchers. Washington, DC: American Psychiatric Press.

[B98] Vaillant G. E. (1995). Adaptation to Life. Cambridge, MA: Harvard University Press.

[B99] Vocks S. Stahn C. Loenser K. Legenbauer T. (2009). Eating and body image disturbances in male-to-female and female-to-male transsexuals. Arch. Sex. Behav. 38, 364–377. 10.1007/s10508-008-9424-z 19030979

[B100] Warrier V. Greenberg D. M. Weir E. Buckingham C. Smith P. Lai M. C.. (2020). Elevated rates of autism, other neurodevelopmental and psychiatric diagnoses, and autistic traits in transgender and gender-diverse individuals. Nat. Commun. 11, 1–12. 10.1038/s41467-020-17794-1 32770077 PMC7415151

[B101] Westen D. Muderrisoglu S. (2003). Reliability and validity of personality disorder assessment using a systematic clinical interview: evaluating an alternative to structured interviews. J. Personal. Disord. 17, 350–368. 10.1521/pedi.17.4.351.23967 14521183

[B102] Westen D. Shedler J. (1999a). Revising and assessing Axis II, Part I: developing a clinically and empirically valid assessment method. Am. J. Psychiatry 156, 258–272. 9989563 10.1176/ajp.156.2.258

[B103] Westen D. Shedler J. (1999b). Revising and assessing Axis II, Part II: toward an empirically based and clinically useful classification of personality disorders. Am. J. Psychiatry 156, 273–285. 9989564 10.1176/ajp.156.2.273

[B104] Witcomb G. L. Bouman W. P. Brewin N. Richards C. Fernandez-Aranda F. Arcelus J. (2015). Body image dissatisfaction and eating-related psychopathology in trans individuals: a matched control study. Eur. Eat. Disord. Rev. 23, 287–293. 10.1002/erv.2362 25944170

[B105] Zimmerman T. N. Porcerelli J. H. Arterbery V. E. (2019). Defensive functioning in cancer patients, cancer survivors, and controls. Psychoanal. Psychol. 36:259. 10.1037/pap0000225

